# Closed Reduction of an Isolated Zygomatic Arch Fracture Under Local Anesthesia: A Case Report

**DOI:** 10.7759/cureus.60987

**Published:** 2024-05-24

**Authors:** Ozan Ates, Gokhan Gocmen, Metin C Kiv

**Affiliations:** 1 Oral and Maxillofacial Surgery, Marmara University, Istanbul, TUR

**Keywords:** ambulatory surgery, closed reduction, maxillofacial trauma, keen technique, isolated zygomatic arch fracture

## Abstract

The zygomatic bone is one of the most prominent bones in the facial region. It forms the most anterolateral projection on each side of the middle face and is articulated with the maxilla, frontal, and temporal bones. Isolated zygomatic arch fractures can occur when a direct force is applied to the zygoma. A variety of intraoral and extraoral techniques have been used as closed reduction techniques for isolated fractures of the zygomatic arch. In this case report, we aim to present our approach for the treatment of a 40-year-old patient with an isolated right zygomatic arch fracture. We used the Keen technique for the closed reduction of the fracture under local anesthesia due to its practicality and effectiveness.

## Introduction

The zygomatic bone is one of the most prominent bones in the facial region. It forms the most anterolateral projection on each side of the middle face and is articulated with the maxilla, frontal, and temporal bones [1]. Zygomatic arch fractures usually occur as part of entire zygomaticomaxillary complex fractures. However, isolated zygomatic arch fractures can occur when a direct force is applied to the zygoma. The incidence of these injuries varies; however, isolated zygomatic arch fractures account for less than 10% of zygomatic injuries [2].

Isolated zygomatic arch fractures generally result in a loss of convexity in the zygomatic arch region [3]. As a result, the normal contours of the temporal area are lost in 57% of isolated zygomatic arch fractures [2]. Trismus occurs due to the impingement of the fractured segment on the temporal muscle, and as a result, limitations of lateral movements to the injured side may be observed. The patients may also present with edema and subconjunctival ecchymosis [3].

A variety of intraoral and extraoral techniques have been used as closed reduction techniques for isolated fractures of the zygomatic arch. The Keen method is an intraoral approach, utilizing a full-thickness maxillary vestibular incision from the canine to the first molar, approximately 5 mm above the mucogingival junction [4].

In this report, we aim to describe a practical and minimally invasive approach for the patient and the surgeon by treating the patient with an isolated zygomatic arch fracture using an intraoral approach under local anesthesia. By performing this procedure intraorally under local anesthesia instead of an extraoral approach under general anesthesia, we aimed to treat the patient cost-effectively, without the risks and associated morbidities.

## Case presentation

**Figure 1:**
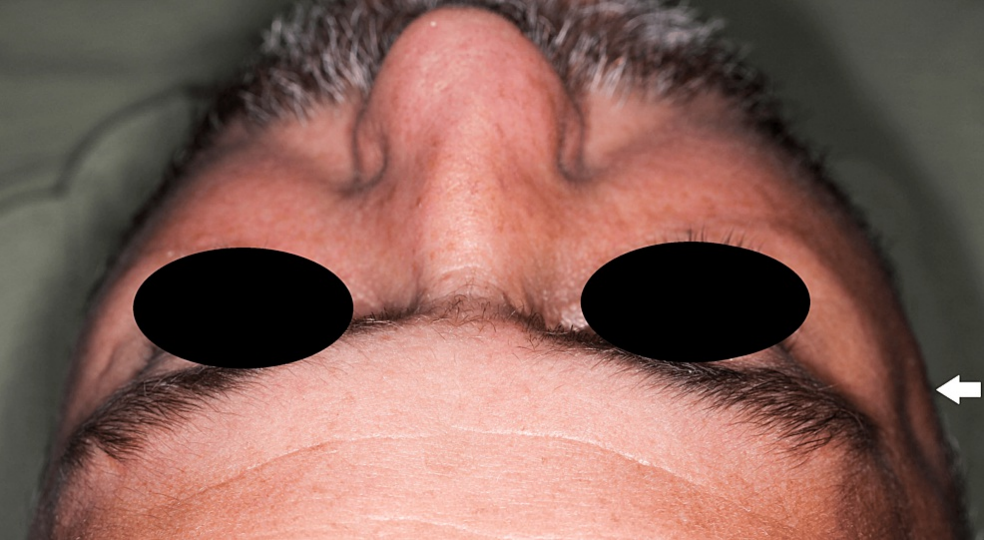
Preoperative extraoral image of the patient

**Figure 2:**
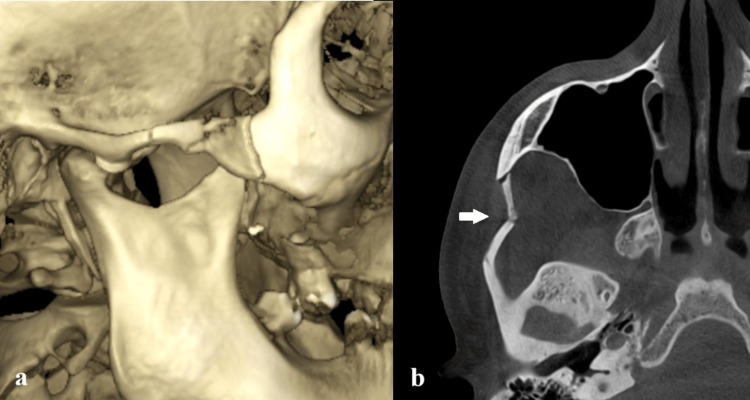
Preoperative dental volumetric tomography images of the patient; (a) the 3D reconstruction of the isolated zygomatic arch fracture; (b) the cone beam computed tomography (CBCT) image revealed a favorable isolated zygomatic arch fracture. The characteristic M-shape of the fractured zygomatic arch can be observed from the axial section of the CBCT.

A 40-year-old patient, with no systemic disease, was referred to our hospital from an external center two days after an alleged history of falling on his right side from a height of about two meters. During the clinical examination, inspection revealed asymmetry and loss of convexity in the right zygomatic arch region, while pain and edema were observed in palpation extraorally (Figure 1). Intraoral inspection revealed that there were no limitations in mouth opening or lateral movements. A cone beam computed tomography image (CBCT) was obtained, and radiologic evaluation revealed a favorable isolated zygomatic arch fracture on the right side (Figure 2). We decided to perform a closed reduction of the zygomatic arch fracture on the day of admission with the Keen technique under local anesthesia due to the favorable nature of the fracture and high patient cooperation.

**Figure 3:**
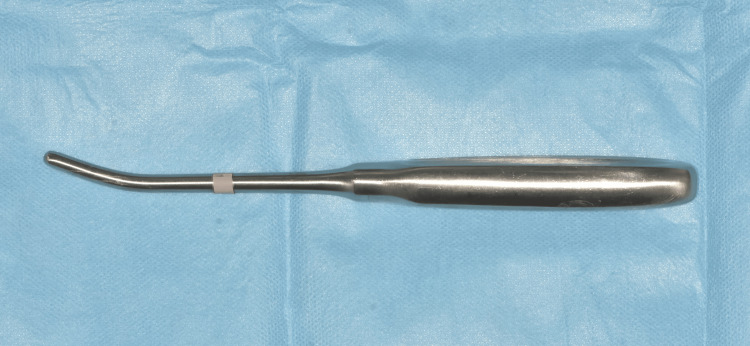
Dingman elevators were used in the closed reduction of isolated zygomatic arch fractures.

**Figure 4:**
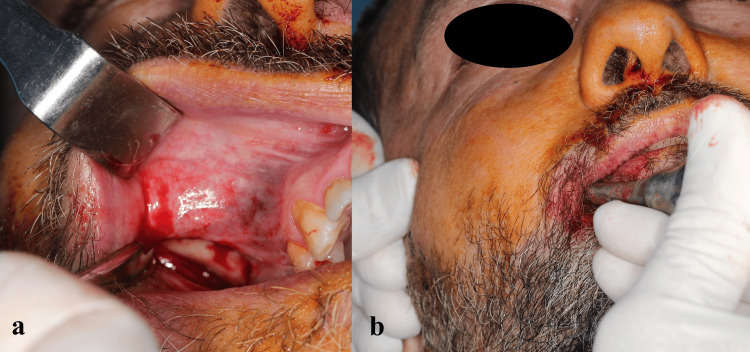
Intraoperative intraoral images of the patient: (a) a 1 cm long vestibular incision is made in the zygomatic arch region; (b) closed reduction is performed with Dingman elevators.

**Figure 5:**
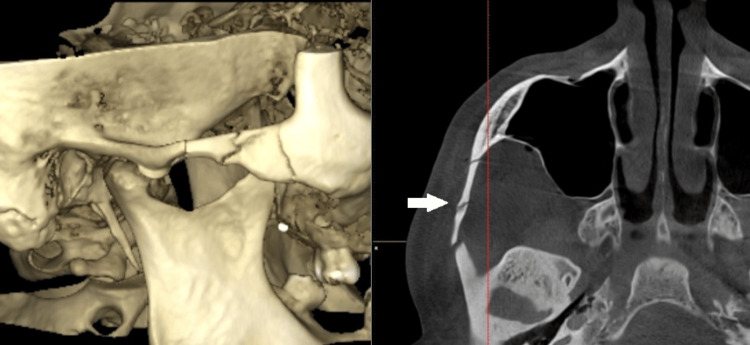
The patient's postoperative dental volumetric tomography image and 3D reconstruction of the fracture

After deep intraoral infiltrative and extraoral infiltrative anesthesia around the zygomatic arch region, a small (≈10 mm) vestibular incision was performed in the vestibular mucosa, just below the zygomatic arch. After the mucoperiosteum was elevated to the zygomatic arch, Dingman elevators (Figure 3) were placed medial to the fracture, and the zygomatic arch fracture was reduced by moving the elevators distally (Figure 4). The patient experienced mild discomfort during the reduction; however, his cooperation was high, and the procedure was not interrupted. After stabilization was achieved, the vestibular incision was closed with 4-0 poly (glycolide-co-lactide) sutures (Pegelak, Doğsan, Turkey). Postoperative CBCT examination showed that the closed reduction of the zygomatic arch fracture was successful, and the zygomatic arch was restored to its natural contours (Figure 5). The patient’s treatment was performed in ambulatory conditions, and the patient was discharged within the procedure day. We prescribed an antibiotic (amoxicillin/clavulanate 1000 mg, 2x1), an anti-inflammatory drug (naproxen 550 mg, 2x1), and an antiseptic mouthwash (chlorhexidine gluconate, 3x1).

**Figure 6:**
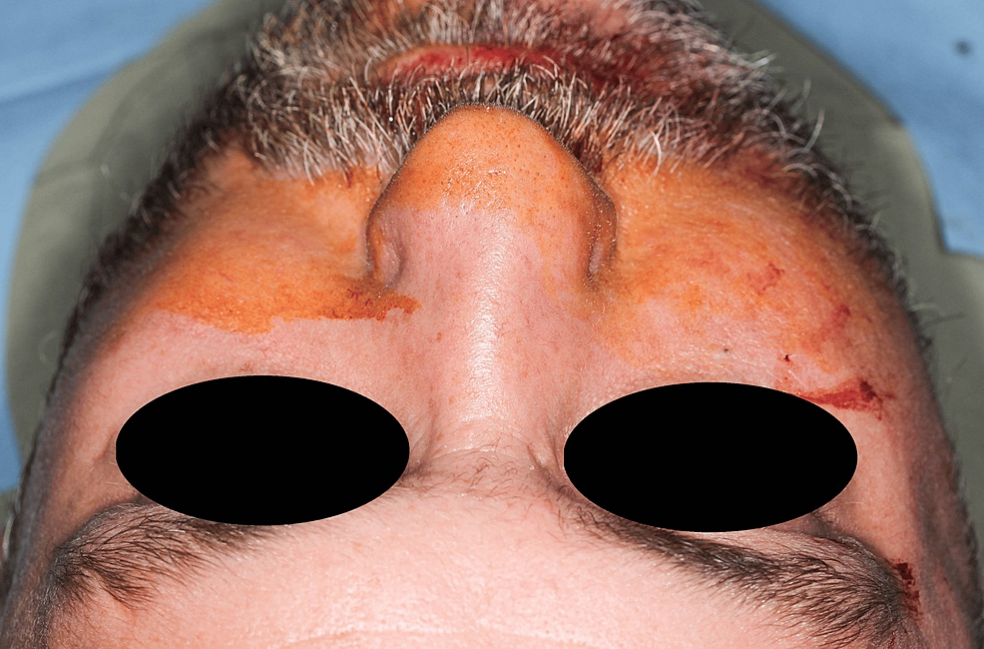
Postoperative extraoral image of the patient; normal convexity is achieved after the procedure.

The patient was called for follow-up in the first postoperative week. There were no symptoms such as restricted mouth opening, pain, or infection (Figure 6). Slightly increased convexity was observed in the right zygomatic arch due to edema. Two weeks later, the patient's asymmetry improved after the resolution of edema, and recovery was uneventful.

## Discussion

Zygomatic arch fractures usually occur as part of entire zygomaticomaxillary complex fractures. However, isolated zygomatic arch fractures can occur when a direct force is applied to the zygoma. The incidence of these injuries varies, with isolated zygomatic arch fractures accounting for less than 10% of zygomatic injuries [2].

Early diagnosis and surgical intervention play a crucial role in the management of zygomatic arch fractures. It is recommended to perform surgical intervention within one week after the injury to optimize patient outcomes [5]. Trauma-related fibrous ankylosis of the temporomandibular joint may be observed in delayed interventions, which results in mouth-opening limitations and thus restricts alternative approaches [6].

Primary evaluation of zygomatic arch fractures may be made via orthopantomogram (OPTG), Water's, and submentovertex radiographs in cases of trauma. The CBCT images are generally needed for further evaluation; zygomatic arch, fracture segments, and asymmetry may be observed clearly in the axial sections [7].

Various approaches for closed reduction have been described in the literature, such as the Keen method, the Gillies method, the Kazanjian method, and the transcutaneous approach in isolated zygomatic arch fractures. We preferred the Keen method since it doesn’t result in extraoral scarring, the facial nerve is protected, and it is a practical procedure performed within a short period.

We also performed the procedure under local anesthesia. This prevented any risk of general anesthesia-related complications and provided a cost-effective and quick approach. In addition, we didn't have to admit our patient and occupy the general anesthesia operating room, which shortened the patient's hospital stay and reduced the related costs. Due to the patient's high level of cooperation, we were able to treat the patient under local anesthesia within the day of admission, in outpatient settings. Since our hospital did not have CBCT in the operating room, we were able to assess the alignment of bony fragments by referring the patient to our oral and maxillofacial radiology department for CBCT evaluation. In addition, performing the surgery under local anesthesia gave us the chance to take a secondary approach and reoperate on the same day. However, we were able to achieve a reduction in our first attempt, and we confirmed it with a postoperative CBCT scan.

## Conclusions

In this case report, we have successfully used the Keen method for the closed reduction of an uncomplicated isolated zygomatic arch fracture under local anesthesia. We think that this method provides the surgeon with a more economical, practical, and safer alternative approach without the general anesthesia-related costs, complications, and morbidity. It also offers the chance to evaluate the procedure in case there is no CBCT in the operating room.
